# Correlates of e-cigarette ad awareness and likeability in U.S. young adults

**DOI:** 10.1186/s12971-017-0125-z

**Published:** 2017-04-04

**Authors:** Jessica M. Rath, Lyubov Teplitskaya, Valerie F. Williams, Jennifer L. Pearson, Donna M. Vallone, Andrea C. Villanti

**Affiliations:** 1 Evaluation Science and Research, Truth Initiative, 900 G. St.,NW, Washington, DC, USA; 2 Department of Health, Behavior and Society, Johns Hopkins Bloomberg School of Public Health, 615 N. Wolf St., Baltimore, MD USA; 3 Zanvyl Krieger School of Arts and Sciences, Johns Hopkins University, 3400 N. Charles St., Baltimore, MD USA; 4 The Schroeder Institute for Tobacco Research and Policy Studies at Truth Initiative, 900 G. St., NW, Washington, DC, USA

**Keywords:** Electronic nicotine delivery devices, Advertising and promotion, Non-cigarette tobacco products

## Abstract

**Background:**

Awareness and use of electronic cigarettes has rapidly increased among U.S. adults. The aim of this study was to examine awareness and likeability of e-cigarette print advertisements in a national sample of young adults and to examine ad likeability as a correlate of intended e-cigarette use among never e-cigarette users.

**Methods:**

Participants (*n* = 2110, unweighted) of the Truth Initiative Young Adult Cohort (January 2013) were randomized to see four print ads (blu, Fin, NJOY, and White Cloud). Bivariate analyses provided descriptive characteristics of all participants and multivariable logistic regression examined the relationships between the average likeability score (across all four ads), curiosity about e-cigarettes, and susceptibility to using e-cigarettes among respondents who had never used e-cigarettes.

**Results:**

Nearly 20% of participants reported awareness of the blu ad. Of the four e-cigarette ads, likeability was highest for the NJOY ad. Participants with higher ad likeability ratings had more than twice the odds of being curious to try an e-cigarette (AOR 2.33; 95% CI 1.84–2.95), try an e-cigarette soon (AOR 2.93; 95% CI 1.96–4.38), and try an e-cigarette if offered by best friend (AOR 2.48; 95% CI 1.95–3.15), after adjusting for other covariates. Current cigarette use was the strongest correlate of susceptibility to using an e-cigarette (*p* < .01) in the multivariable models.

**Conclusions:**

Higher ad likeability was correlated with greater susceptibility to try an e-cigarette among U.S. young adults. Future studies are needed to monitor how awareness and likeability of e-cigarette advertising influence patterns of e-cigarette and other tobacco use in young people.

## Background

Awareness of electronic cigarettes (e-cigarettes) has rapidly increased among United States (U.S.) adults ages 18 and over from 40.9% in 2010 to 79.7% in 2013 [1]. Ever and past 30-day e-cigarette use in adults have increased during this time as well [1, 2]. This may be driven, in part, by experimentation in young adults, ages 18–24, of this adult population [3]. In a 2014 national sample of adults, ever use of e-cigarettes was correlated with daily cigarette smoking, white race, younger age (age 18–24), and living in the Western U.S. [3] The same study found that young adults (18–24) were more likely to be some day or every day e-cigarette users than were adults over age 45 [3]. High current levels of e-cigarette awareness in the young adult population may be explained, in part, by an increased exposure to e-cigarette advertising and promotions in the U.S.

Advertising plays an important role in raising awareness of novel products and has been shown to influence product initiation and facilitate progression to regular use in youth [3, 4]. With the entry of three of the top four tobacco manufacturers, Philip Morris, Reynolds American, and Lorillard, into the e-cigarette market, spending on e-cigarette advertising increased dramatically after 2010 [5, 6]. Advertising expenditures totaled $22 million in 2012 [5, 6] and increased to $115.3 million in 2014 [6]. The largest amount of e-cigarette marketing expenditures has been allocated to magazines (over $83 million in 2014), followed by cable television [6]. At the time of the current study, in 2013, blu e-cigarettes was the most promoted brand, comprising 60% (over $14 million) of total promotional expenditures [5].

Although the link between advertising exposure, likeability of cigarette advertising, and cigarette use has been well established in the literature [7], there is limited empirical evidence on the relationship between advertising exposure and likeability of e-cigarette advertising. Advertisement likeability is a strong predictor of ad campaign success [8, 9]. Evidence from three studies suggests that exposure to e-cigarette advertisements is increasing over time [6, 10, 11]. One study indicated that 76% (22.7 million) of U.S. young adults were exposed to e-cigarette print ads in 2013 [12]. Another study demonstrated that young adult exposure to television e-cigarette advertisements increased 321% from 2011 to 2013 [10]. Among a sample of 307 college students, 90% of students reported being exposed to some form of e-cigarette marketing “sometimes” or “often” [11]. Lifetime e-cigarette users reported slightly higher exposure to e-cigarette marketing and were more likely to like e-cigarette advertising compared to non-users, though it is unclear whether exposure to e-cigarette advertising pre- or post-dated e-cigarette use in this study [11]. Limited evidence suggests that exposure to e-cigarette television [13] and print advertisements [14] may increase curiosity and intention to try these novel products in a small proportion of young people.

Advertising of e-cigarettes occurs predominantly through channels that appeal to young people, particularly where marketing of other tobacco products is banned (e.g., television, sponsorships) [6, 15]. Despite the evidence surrounding tobacco advertising exposure and cigarette smoking behavior in youth and young adults [4, 7], evidence on the impact of e-cigarette advertising on e-cigarette experimentation and progression to more regular use among youth and young adults is nascent [13, 14]. From a regulatory perspective, it is also important to consider the impact of e-cigarette advertising on the overall U.S. population as outlined in the public health standard that guides Food and Drug Administration’s (FDA) regulatory actions on tobacco [16, 17]. This includes the potential ways in which exposure to e-cigarette advertising affects patterns of use of other tobacco products [18], and combustible tobacco that causes the overwhelming majority of preventable deaths [4]. Villanti et al. study in a national sample of young adults showed that brief exposure to four e-cigarette ads increased curiosity and trial of e-cigarettes, but did not examine whether the ads themselves were appealing or how ad appeal might have impacted curiosity and susceptibility to use e-cigarettes [19]. The purpose of this study is to provide more detail and context for the initial trial findings by examining factors related to awareness and likeability of four e-cigarette print advertisements and ad likeability as a correlate of intended e-cigarette use among a national sample of young adults that have never used e-cigarettes. We hypothesized that ad likeability would predict curiosity and susceptibility to use e-cigarettes.

## Methods

The present study used data from the Truth Initiative Young Adult Cohort Study which was designed to understand the trajectories of tobacco use in a young adult population. Briefly, the cohort was comprised of a nationally representative sample of young adults ages 18-34 drawn from GfK’s KnowledgePanel®. KnowledgePanel® is an online panel of adults ages 18 and older that covers both the online and offline populations in the U.S. The panel was recruited via address-based sampling, a probability-based random sampling method that provides statistically valid representation of the U.S. population, including cell phone-only households. The validity of this methodology has been reported previously [20, 21], and KnowledgePanel® samples have been used broadly in studies in the peer-reviewed medical literature [22–25]. The detailed methods of this study have been described elsewhere [26].

Data for the longitudinal study is collected every six months. This analysis used data from Wave 4, collected at approximately year two of the study, because all respondents in Wave 4 (*n* = 4,288) were involved in a randomized controlled trial on e-cigarette advertising [14]. These data were collected in January 2013. The panel recruitment rate (RECR) [27] for Wave 4 was 14.7%. In 65.5% of these households, one member completed a core profile survey in which the key demographic information was collected (profile rate—PROR). One panel member per household was randomly selected to be part of the study sample and no members outside the panel were recruited. The completion rate (COMR) was 65.7%. Thus, the product of these three rates, the cumulative response rate (CUMRR1), was 6.3%. This study was approved by the Chesapeake Institutional Review Board, Inc., and online consent was collected from participants before survey self-administration.

All respondents participating in the Wave 4 survey (*N* *N* = 2110 unweighted) were involved in the trial and were randomized in a 1:1 ratio to one of two conditions: exposure to four different e-cigarette ads (blu, Fin, NJOY, White Cloud) (*n* = 2110) or no ad exposure (*n* = 2178). These ads were chosen from a comprehensive advertising surveillance system (Competitrack; www.competitrack.com) to have a similar level of production quality. Data from Competitrack indicate that three of the ads were presented in print media (blu, Fin, NJOY), with the fourth presented in an online display (White Cloud). In 2012, the blu ad had the largest reach with 18 insertions and an estimated total spend of $1,730,800, followed by the NJOY ad (estimated $327,700 spend for 3 insertions), the Fin ad (estimated $124,700 spend for 1 insertion), and White Cloud (estimated $392 spend for 11 days on a website). Randomization was accomplished using a sequence generated within the survey software that was not accessible to investigators or participants, thus maintaining allocation concealment. The order of the ads was not randomized within the exposed group. The ads used in the trial were selected from a comprehensive advertising tracking system (Competitrack; www.competitrack.com) and were presented as screen grabs from the actual advertisements to participants. The blu ad had the largest reach in 2012 with a total spend of $1,730,800 for 18 insertions followed by the NJOY ad (3 insertions for $327,700), the Fin ad ($124,700 spend for 1 insertion), and White Cloud ($392 spend for 11 days on a website). Methodologic details of this trial are presented elsewhere [14].

### Measures

#### Outcomes

Outcome measures included awareness of the advertisements, curiosity about e-cigarettes, and openness to use e-cigarettes in the future. Awareness of the advertisement was assessed by asking “Have you seen this advertisement before?” with binary response choices (yes/no).

The item “Have you ever been curious about smoking e-cigarettes?” was used to assess curiosity about e-cigarettes (yes/no). Two items were used to assess susceptibility (likelihood of future use) to e-cigarettes including “Do you think that you will try an e-cigarette soon?” and “If one of your best friends were to offer you an e-cigarette, would you try it?” These two items are based on measures of susceptibility to cigarette smoking among adolescents [28, 29] and had the following response choices: “Definitely yes,” “Probably yes,” “Probably not,” and “Definitely not.” Results of exploratory analyses showed that there were few differences between probably and definitely responses, so each item was treated as a dichotomous variable: probably not/definitely not or definitely/probably yes. Analyses including these outcomes focused on the subset of respondents who had never used an e-cigarette (*n* = 1952).

### Covariates

Smoking status: Cigarette smoking and e-cigarette use were determined using two items asking about ever use and past 30-day use. Ever use of either an e-cigarette or a cigarette was assessed at Wave 4 and defined as any prior use of an e-cigarette or cigarette, even a puff, respectively. Current use was defined as any use in the past 30 days and non-current users were defined as those who smoked on 0 days of the last 30 or had never smoked a cigarette.

Other influences on smoking: Peer smoking and exposure to other tobacco advertising in the past six months were examined as other possible influences on e-cigarette use. Peer smoking was evaluated using the following item: “How many of your 4 closest friends smoke cigarettes?” with respondents entering a number between 0 and 4. The responses were dichotomized as “0” and “1 or more.” Exposure to tobacco advertising was assessed by asking “In the past 6 months, have you done any of the following? Select all that apply” with the following response choices: 1. “Visited and/or registered on a tobacco company or product website,” 2. “Visited, friended or otherwise engaged with a Facebook or other social media page dedicated to a tobacco product,” 3. “Been exposed to and/or participated in a tobacco product event at a festival, concert, bar or clubs,” and 4. “Received direct mail or email advertising tobacco products.”

Other control variables: Ad likeability, which has strong predictive power for advertising success [8, 9], was assessed by asking participants to describe their feelings about each ad, with responses on a 5-point scale coded from “I disliked it very much” to “I liked it very much.” An average likeability score for each participant was computed across the four ads (range 1–5) for use in the multivariable models. E-cigarette product awareness was assessed by asking, “Have you ever heard of a product called an electronic cigarette or e-cigarette or brands such as Smoking Everywhere, NJOY, Gamucci, or others?” Sociodemographic items assessed included age (grouped as 18–24 and 25–34), gender, educational attainment (less than high school, high school, and some college or greater), ratio of household income to 2012 poverty threshold (less than 1, greater than or equal to 1) and race/ethnicity (White, non-Hispanic; Black, non-Hispanic; Other, non-Hispanic; and Hispanic). The Other, non-Hispanic category included Asians, Pacific Islanders, Native Americans, Native Alaskans and respondents who self-identified as multiracial.

### Data analysis

All analyses were performed using Stata/SE 13.1 (StataCorp 2014) and post-stratification weights were used to offset any non-response or non-coverage bias and produce nationally representative estimates. Chi-square tests were used to assess associations between categorical covariates and the outcome variables and *t* tests were used to examine differences between mean likeability scores by ad. The Benjamini-Hochberg procedure was used in the bivariate analysis to correct for multiple comparisons (FDR = 0.1) [30]. Multivariable logistic regression was used to examine the relationships between the average likeability score (mean across all four ads), curiosity about e-cigarettes, and susceptibility to using e-cigarettes among respondents who reported never having used e-cigarettes before. These analyses controlled for age, sex, race/ethnicity and education as well as for covariates associated with the secondary outcomes in the bivariate analyses (income to poverty ratio, current cigarette use, having ever heard of e-cigarettes, peer smoking, and other ad experiences). *P*-values associated with the *t* statistic were used to identify significant correlates of likeability.

## Results

### Participant characteristics

The study sample was comprised of only those exposed to an e-cigarette ad. 2,110 young adults (unweighted) aged 18–34 years were shown the ads during the study. The weighted sample size was 2,093 with 42.4% aged 18–24 years (Table 1). About 59% of the sample was non-Hispanic White, with 12.6% non-Hispanic Black, 19.2% Hispanic, and 9.6% non-Hispanic “other” race. The majority (61.2%) of participants had at least some college education, and 75.1% of participants lived above the federal poverty line. Among young adults in the sample, 6.8% had ever used an e-cigarette, and 2.0% had used an e-cigarette in the past 30 days. Though 20.4% of participants reported smoking in the past 30 days, only 10.9% described themselves as smokers, with an additional 11.3% describing themselves as social or occasional smokers, 8.3% as ex-smokers, 8.1% as someone who had tried smoking, and 61.4% as non-smokers. A little more than half (54.3%) of respondents reported that one or more of their four closest friends smoke cigarettes. The prevalence of engagement with tobacco marketing ranged from 4.1% ever visiting a tobacco company website to 18.4% receiving tobacco product direct mail or email advertisements. Of the total sample, 18.4% reported curiosity to try an e-cigarette, while 8.1% would try an e-cigarette soon and 20.2% would try an e-cigarette if their best friend offered it to them. No differences were observed at the *p* < .01 level in the three outcome measures by age group (Table 1).

**Table 1:** Participant characteristics (*n* = 2093). WEIGHTED

Sociodemographic characteristics	Percent
Gender	
Male	49.1
Female	50.9
Age	
18–24	42.4
25–34	57.6
continuous (mean, SD)	25.9, 5.07
Race/ethnicity	
White, non-Hispanic	58.6
Black, non-Hispanic	12.6
Other, non-Hispanic	9.6
Hispanic	19.2
Education	
Less than high school	11.3
High school	27.5
Some college or more	61.2
Income to poverty^a^ ratio 1+	75.1
Exposure to other tobacco advertising	
Visited/registered on tobacco company website	4.1
Engaged with a tobacco social media page	5.2
Exposed to/participated in tobacco product event	10.2
Received direct mail/email tobacco product ads	18.4
Tobacco-use related items	
Current cigarette use^b^	20.4
Ever e-cigarette use	6.8
Current e-cigarette use^b^	2.0
Intent-related among e-cigarette never users	
Ever curious about smoking e-cigarettes	18.4
Will try an e-cigarette soon	8.1
Would try an e-cigarette if offered by a friend	20.2
Self-identified smoking status	
Smoker	10.9
Social/occasional smoker	11.3
Ex-smoker	8.3
Tried smoking	8.1
Non-smoker	61.4
Peer smoking--none vs ≥ 1 of closest friends smoke cigarettes 0/1	54.3

^a^using 2012 poverty guidelines

^b^Current user defined as used product one or more days in past 30 days

### Awareness of selected e-cigarette ads

The greatest percentage of participants expressed awareness of the blu e-cigarette ad (19.2%), followed by the NJOY (8.8%), the White Cloud (4.4%), and FIN ad (2.0%) (Table 2). No significant differences in awareness of any of the ads were observed by age group or gender. A significantly greater proportion of non-Hispanic Blacks (34.0%; *p* < .001) reported having seen the blu e-cigarette ad as compared to participants of other races/ethnicities. Hispanics had the lowest prevalence of awareness of the blu ad (13.5% *p* = .024) relative to participants of other races/ethnicities. Significantly lower proportions of non-Hispanic Whites had seen the NJOY (5.9%; *p* < .001) and White Cloud ads (2.2%; *p* = .001) compared to respondents of other races/ethnicities. Those with a high school education had the highest prevalence of awareness of the White Cloud ad (8.3%; *p* = .001) relative to respondents who had not completed high school or had completed some college or more. More current cigarette smokers expressed awareness of the blu ad compared to non-current smokers (27.7% and 16.9%; *p* = .006). There was a significant positive association between awareness of the blu, NJOY, or FIN ad and having one or more of four closest friends who smoke (*p* < .001, *p* = .022, and *p* < .001, respectively). Significantly more of the respondents who engaged with a tobacco social media page reported awareness of the White Cloud and FIN ads relative to those respondents who had not engaged with such a media page (*p*s < .001). Also, participants who reported having received tobacco product ads via direct mail or email were significantly more likely to express awareness of the NJOY and blu ads (*p* = .003 and *p* = .001, respectively) (Table 2).

**Table 2:** Awareness of advertisements (*n* = 2,093) WEIGHTED

	Previously Saw Advertisement^a^			
	White Cloud	NJOY	FIN	Blu
Overall (% of total)	4.4	8.8	2.0	19.2
Age Group (%)				
18–24	4.7	9.9	1.7	19.8
25–34	4.3	8.0	2.2	18.7
Gender (%)				
Male	4.1	7.9	1.1	19.9
Female	4.7	9.6	2.8	18.5
Race/ethnicity (%)				
White, non-Hispanic	2.2**	5.9**	1.2	17.5
Black, non-Hispanic	5.5	13.5	1.6	34.0**
Other, non-Hispanic	9.2	14.2	6.7**	21.0
Hispanic	8.0*	11.7	2.3	13.5*
Education (%)				
Less than high school	2.6	11.0	1.3	15.2
High school	8.3**	9.6	3.1	20.9
Some college or more	3.0*	8.0	1.6	19.1
Income to poverty ratio (%)				
Ratio 1+	3.4	8.0	2.0	18.1
Ratio ≤ 1	7.6*	11.2	1.8	22.5
Current Tobacco Use (%)				
Current cigarette use^b^	3.4	11.4	2.6	27.7**
No current cigarette use	4.7	8.0	1.8	16.9
Current e-cigarette use^b^	4.8	8.9	0.8	29.5
No current cigarette use	4.4	8.8	2.0	19.0
Other ad experiences (%)				
Visited/registered on a tobacco co. website	9.6	14.7	2.9	38.9**
Engaged with a tobacco social media page	25.1**	17.9	15.1**	31.7
Exposed to/participated in tobacco product event	8.6	9.9	2.5	27.2
Received direct mail/email tobacco product ads	3.4	15.0**	3.9	28.5**
Peer smoking (%)				
One or more of closest friends smoke	5.7	10.9*	3.4**	25.0**
None of closest friends smoke	2.9	6.1	0.3	12.0
E-cigarette awareness				
Ever heard of e-cigarettes	4.6	10.6**	2.4	23.2**
Never heard of e-cigarettes	4.0	3.9	0.9	9.5
Ad likeability mean(SD)	2.7(1.1)	2.9*(1.2)	2.5*(1.2)	2.7(1.2)

*adjusted *p* < .05

**adjusted *p* < .01

^a^Seen ad—White Cloud *n* = 92, NJOY *n =* 181, FIN *n* = 41, blu *n* = 396; for variables with three or more categories, *p* values are from the chi-square statistic calculated for column percentages (e.g., WNH compared to non-WNH); for ad likeability, scores were compared to each other and *p* values are from the t statistic

^b^One or more days of use in past month

### Susceptibility to e-cigarette use

Current cigarette smokers (*p* < .001), those who had visited or registered on a tobacco company website (*p* < .001), those who received direct mail or email tobacco product ads (*p* < .006), those who had ever heard of e-cigarettes (*p* < .01) and those with one or more close friends who smoke (*p* < .01) were more likely to report openness to e-cigarette use. Participants who had engaged with a tobacco social media page were more likely to report intention to try an e-cigarette soon (*p* = .001) (data not shown in table).

### Likeability of selected e-cigarette ads

Table 2 indicates that of the four e-cigarette ads, participants reported the highest mean likeability score for the NJOY ad (2.9; standard deviation [SD] 1.2), which featured the tagline, “The most amazing thing about this cigarette? It isn’t one,” followed by blu (2.7; SD 1.2), White Cloud (2.7; SD 1.2), and FIN (2.5; SD 1.2; significantly different from NJOY *p* = .042).

Results of multivariable logistic regression analysis among the subset of cases who had never tried an e-cigarette (*n* = 1952) are presented in Table 3. Analyses controlling for age, gender, race, education and for covariates associated with the secondary outcomes in the bivariate analyses (income to poverty ratio, other ad experiences, current cigarette use, having ever heard of e-cigarettes and peer smoking) revealed that, for each one-point increase in likeability rating, the odds of reporting being curious to try an e-cigarette was more than twice as high (AOR 2.33; 95% CI 1.84–2.95) for any of the ads. Similar relationships were observed between likeability rating and being open to trying an e-cigarette soon (AOR 2.93; 95% CI 1.96–4.38) and trying if offered an e-cigarette by best friend after adjusting for covariates (AOR 2.48; 95% CI 1.95–3.15). In all three of the multivariable models, current cigarette use and peer smoking were the strongest positive correlates of susceptibility to using an e-cigarette (all *p* < .01). Other, non-Hispanic race/ethnicity was a strong correlate of two of the three outcome measures—curiosity to try an e-cigarette and would try an e-cigarette if good friend offered (*p* < .01). An additional interaction analysis demonstrated that there were no significant interactions between current cigarette use or peer smoking and the outcomes of interest.

**Table 3:** Ad likeability as a correlate of intended e-cigarette use among never users (total weighted *n* = 1,952)

	Curiosity to try an e-cigarette (*n* = 1909)		Intent to try an e-cigarette soon (*n* = 1898)		Would try e-cigarette if best friend offered (*n* = 1900)	
	Adj OR	(95% CI)	Adj OR	(95% CI)	Adj OR	(95% CI)
Ad likeability	2.33**	(1.84–2.95)	2.93**	(1.96–4.38)	2.48**	(1.95–3.15)
Gender						
Male	Ref		Ref		Ref	
Female	0.77	(0.51–1.15)	0.79	(0.43–1.47)	0.58	(0.39–0.88)
Age group						
18–24	1.34	(0.86–2.08)	1.07	(0.52–2.20)	1.08	(0.67–1.73)
25–34	Ref		Ref		Ref	
Race/ethnicity						
White, non-Hispanic	Ref		Ref		Ref	
Black, non-Hispanic	1.14	(0.53–2.42)	1.12	(0.41–3.10)	0.98	(0.45–2.11)
Other, non-Hispanic	3.31**	(1.63–6.73)	2.97	(0.91–9.689	3.07**	(1.53–6.15)
Hispanic	1.04	(0.57–1.87)	1.71	(0.74–3.87)	1.03	(0.55–1.91)
Education level						
Less than high school	Ref		Ref		Ref	
High school	0.95	(0.44–2.06)	0.57	(0.21–1.56)	1.17	(0.53–2.59)
Some college or more	1.55	(0.74–3.25)	0.68	(0.26–1.80)	1.40	(0.62–3.13)
Income to poverty ratio						
Ratio < 1	0.75	(0.43–1.31)	0.90	(0.43–1.88)	0.71	(0.40–1.25)
Ratio ≥ 1	Ref		Ref		Ref	
Other ad experiences						
Visited/registered on tobacco company website	2.71	(0.89–8.27)	1.22	(0.34–4.39)	2.78	(0.72–10.79)
Engaged with a tobacco social media page	2.00	(0.83–4.78)	1.85	(0.63–5.47)	0.90	(0.33–2.51)
Received direct mail/email tobacco product ads	0.69	(0.37–1.29)	1.18	(0.56–2.47)	0.65	(0.33–1.28)
Current cigarette use (No = ref)						
Yes	8.01**	(4.96–12.97)	12.69**	(6.34–25.41)	18.96**	(11.43–31.45)
Ever heard of e-cigarettes (No = ref)						
Yes	1.67*	(1.03–2.73)	1.25	(0.56–2.76)	2.03**	(1.22–3.38)
Peer smoking (None of closest friends smoke = ref)						
One or more of closest friends smoke	2.37**	(1.47–3.82)	7.15**	(2.43–21.03)	2.84**	(1.73–4.666)

*p < .05; **p < .01

## Discussion

This study provides early data on awareness and likeability of e-cigarette advertising and associated factors among young adults. Results also provide some evidence of the potential influence of ad likeability on curiosity and intention to try these products. While the strongest correlates of curiosity and intention to use e-cigarettes were current cigarette smoking and peer cigarette smoking in all three multivariable models, ad likeability remained associated with a more than two-fold increase in the odds of curiosity and intention to use e-cigarettes among never users.

This study also identified differences in likeability in four selected e-cigarette ads and the potential impact of ad likeability on intentions to use e-cigarette products. In this study, participants liked the NJOY ad more than the other 3 ads. For all 4 ads, those with more positive likeability had significantly greater curiosity to try an e-cigarette. This finding may provide some explanation for why ad exposure increased curiosity in the ad experiment and part of the mechanism linking curiosity to e-cigarette trial in our other study. In the ad experiment, among young adults who had never used an e-cigarette, 14.8% reported that they were curious to try an e-cigarette, with a greater percentage of the exposed (18.4%) versus unexposed group (11.3%) reporting curiosity [14]. Higher likeability ratings were also associated with greater likelihood of being open to trying an e-cigarette soon and trying one if offered by a best friend (20% of the sample). Measures of exposure to marketing that capture likeability or receptivity are more likely related to present or future smoking behavior than are measures of external exposure [7].

Not surprisingly, the blu ads were reported as the most widely seen. This finding is consistent with multiple studies which confirm blu as the most advertised e-cigarette at this time in the U.S. market [5, 6, 10, 31]. More specifically, in a study highlighting that blu ads represented over 80% of the e-cigarette ads, findings showed that non-Hispanic Blacks had a greater prevalence of having seen blu e-cigarette ads than participants of other races and ethnicities [10]. At the time of this study, blu was owned by Lorillard, which had a long history of targeted marketing to racial/ethnic minority youth and young adults [4, 32–34]. Despite rapid changes in the e-cigarette marketplace, our findings from 2012 data remain relevant based on advertising data. In 2014, the top five brands marketed were blu, MarkTen, NJOY, Vuse, and Fin and magazine advertising accounted for 72% of advertising expenditures [6]. Three of the four brands represented in this study (blu, NJOY, Fin) remained highly marketed in 2014 where print ads in magazines still represented the dominant form of e-cigarette advertising.

There are several limitations to this study. First, the exposure to static e-cigarette ad images in this study does not adequately reflect the exposure experienced by young adults in the real world. Second, the ads selected for this study do not represent the possible range of e-cigarette advertising which may also include radio, television, internet, and advertisements at the point-of-sale. Therefore, the findings of this study may overestimate or underestimate the true effect of this type of advertising on curiosity, trial and established use. Third, the cross-sectional nature of the study only allows us to examine correlations and does not allow us to examine the temporal relationship between ad likeability and curiosity or intention to use e-cigarettes. Fourth, some limited sample sizes exist for two subgroups for which we find significant results: those reporting being open to try an e-cigarette soon (8%) and those reporting being curious to try an e-cigarette (18%). Finally, this study employs an existing online panel to recruit a large, nationally representative cohort of young adults, a group typically identified as hard-to-reach. The study sample’s completion rate (65.7%) and cumulative response rate (6.3%) are similar to that of other health studies that have relied on KnowledgePanel [23–25, 35]. The internal validity of our results is not compromised by the panel’s cumulative response rate and other work suggests that surveys with a low response rate can still be representative of the sample population, even though the risk of nonresponse bias is higher [36, 37]. Studies assessing nonresponse to panel recruitment in KnowledgePanel have found little indication of nonresponse bias on core demographic and socioeconomic variables [38, 39] and previous estimates from this cohort for key outcomes of interest, such as ever and current cigarette use, are consistent with national survey data [26].

## Conclusion

Higher ad likeability was correlated with greater susceptibility to try an e-cigarette among U.S. young adults. In terms of the public health standard, it is important to determine the net impact of e-cigarette advertising within the overall context of tobacco use in youth, young adults, and adults, as well as potential benefits and harms to the population [18]. Exposure to e-cigarette advertising could facilitate uptake, cessation, or have no effect on patterns of e-cigarette and tobacco use. Future studies are needed to consistently monitor how awareness and likeability of e-cigarette advertising may influence patterns of use for youth and young adults, especially in terms of use of other tobacco products and combustible products (cigarettes, cigars, hookah, pipe, bidis, and roll your own). Studies also need to examine the impact of content, themes, channels, and other ad features that may prompt e-cigarette curiosity, trial, and progression to or cessation of other tobacco product use.

## Abbreviations

AOR: Adjusted odds ratios
CI: Confidence intervals
COMR: Completion rate
CUMRR1: Cumulative response rate
e-cigarettes: Electronic cigarettes
FDA: Food and drug administration
PROR: Profile rate
RERC: Panel recruitment rate
U. S: United States
